# Human T-Lymphotropic Virus Type 1 transcription and chromatin-remodeling complexes

**DOI:** 10.1186/1742-4690-8-S1-A178

**Published:** 2011-06-06

**Authors:** Rebecca Easley, Lawrence Carpio, Irene Guendel, Zachary Klase, Soyun Choi, Kylene Kehn-Hall, Fatah Kashanchi

**Affiliations:** 1 George Mason University, Department of Molecular and Microbiology, National Center for Biodefense and Infectious Diseases, Manassas, VA, 20110, USA; 2 National Institutes of Health, Molecular Virology Section, Laboratory of Molecular Microbiology, NIAID, Bethesda, Maryland, 20892, USA; 3 The George Washington University Medical Center, Department of Microbiology, Immunology, and Tropical Medicine, Washington, DC, 20037, USA

## 

Human T-lymphotropic virus type 1 (HTLV-1) encodes the viral protein Tax, which is believed to act as a viral transactivator through its interactions with a variety of transcription factors, including CREB and NF- B. As is the case for all retroviruses, the provirus is inserted into the host DNA, where nucleosomes are deposited to ensure efficient packaging. Previous studies using immunoprecipitation from Tax-expressing cells, 2-dimensional gel electrophoresis, and mass spectrometry analysis have identified the ATPase subunit BRG1 as a Tax-interacting protein. BRG1, is part of at least 8 complexes which include BAF and PBAF, as well as WINAC, NCoR, mSin3A/HDAC, and NUMAC. Nucleosomes act as roadblocks in transcription, making it difficult for RNA polymerase II (Pol II) to proceed toward the 3' end of the genome. Our results using HTLV-1 integrated chromatin indicate that the Tax-activated promoter utilized PBAF complex which appears to be responsible for nucleosome remodeling. Also, the promoter was assembled into an ordered array of translationally positioned nucleosomes. In contrast, the inactive promoter was relatively inaccessible to nuclease and was not assembled into a translationally positioned nucleosomal arrays.

